# Rapid Antigen Detection Tests for Malaria Diagnosis in Severely Ill Papua New Guinean Children: A Comparative Study Using Bayesian Latent Class Models

**DOI:** 10.1371/journal.pone.0048701

**Published:** 2012-11-05

**Authors:** Laurens Manning, Moses Laman, Anna Rosanas-Urgell, Berwin Turlach, Susan Aipit, Cathy Bona, Jonathan Warrell, Peter Siba, Ivo Mueller, Timothy M. E. Davis

**Affiliations:** 1 School of Medicine and Pharmacology, University of Western Australia, Fremantle Hospital, Fremantle, Western Australia, Australia; 2 Papua New Guinea Institute of Medical Research, Madang, Papua New Guinea; 3 Centre for Applied Statistics, University of Western Australia, Crawley, Western Australia, Australia; 4 Barcelona Centre for International Health Research (CRESIB), Barcelona, Spain; 5 Walter and Eliza Hall Institute, Parkville, Melbourne, Australia; Pennsylvania State University College of Medicine, United States of America

## Abstract

**Background:**

Although rapid diagnostic tests (RDTs) have practical advantages over light microscopy (LM) and good sensitivity in severe falciparum malaria in Africa, their utility where severe non-falciparum malaria occurs is unknown. LM, RDTs and polymerase chain reaction (PCR)-based methods have limitations, and thus conventional comparative malaria diagnostic studies employ imperfect gold standards. We assessed whether, using Bayesian latent class models (LCMs) which do not require a reference method, RDTs could safely direct initial anti-infective therapy in severe ill children from an area of hyperendemic transmission of both *Plasmodium falciparum* and *P. vivax.*

**Methods and Findings:**

We studied 797 Papua New Guinean children hospitalized with well-characterized severe illness for whom LM, RDT and nested PCR (nPCR) results were available. For any severe malaria, the estimated prevalence was 47.5% with RDTs exhibiting similar sensitivity and negative predictive value (NPV) to nPCR (≥96.0%). LM was the least sensitive test (87.4%) and had the lowest NPV (89.7%), but had the highest specificity (99.1%) and positive predictive value (98.9%). For severe falciparum malaria (prevalence 42.9%), the findings were similar. For non-falciparum severe malaria (prevalence 6.9%), no test had the WHO-recommended sensitivity and specificity of >95% and >90%, respectively. RDTs were the least sensitive (69.6%) and had the lowest NPV (96.7%).

**Conclusions:**

RDTs appear a valuable point-of-care test that is at least equivalent to LM in diagnosing severe falciparum malaria in this epidemiologic situation. None of the tests had the required sensitivity/specificity for severe non-falciparum malaria but the number of false-negative RDTs in this group was small.

## Introduction

The World Health Organization (WHO) advocates treatment of malaria based on universal access to light microscopy of blood smears (LM) and/or antigen-based rapid diagnostic tests (RDTs) [1]. LM has been the conventional reference method but requires trained technicians and good quality smears, and has limited sensitivity for low parasite densities and mixed *Plasmodium* species [2]. RDTs can be performed efficiently and accurately with minimal training. Most RDTs have sensitivities and specificities of 85% to 95% for *P. falciparum* in a variety of settings [3], [4], but lower detection rates and sensitivities for non-falciparum malaria [4]. A limitation of such studies has been the shortcomings of LM as a reference method [5]. When LM and RDTs have been compared with more sensitive polymerase chain reaction (PCR)-based methods, RDTs often outperform LM for the detection of *P. falciparum* [6], [7] but not *P. vivax* [8]. However, the nucleic acids detected by PCR may be from non-viable parasites or gametocytes. Therefore, in studies of malaria diagnostic modalities, the comparators can only ever be as good as, but never better than, an imperfect gold standard.

The currently recommended application of malaria diagnostic tests depends on the clinical situation. PCR-based methods are not yet in routine field use. Quality-assured LM and RDTs are considered equivalent in uncomplicated malaria [5], [9]. In suspected severe malaria, a presumptive course of antimalarial and antibiotic treatment is recommended regardless of the results of diagnostic tests which are commonly performed or reported after initiation of management [10]. LM is preferred over RDTs in this situation because parasite density can guide adjunctive therapy and facilitate monitoring of the response to treatment [1]. Although universal antimalarial therapy avoids the potentially dire consequences of a false negative result, inappropriate use of artemisinin-based therapy may promote parasite resistance [11], while there may be adverse outcomes when artemisinin therapy is given to children with meningeal inflammation [12]. Over-reliance on empiric therapy may also delay diagnosis and treatment of other life-threatening infections with consequently increased mortality [13]. In addition, an accurate initial diagnosis and rational treatment reduces costs associated with broad-spectrum presumptive anti-infective therapy, a particular benefit in resource-poor settings.

**Figure 1:**
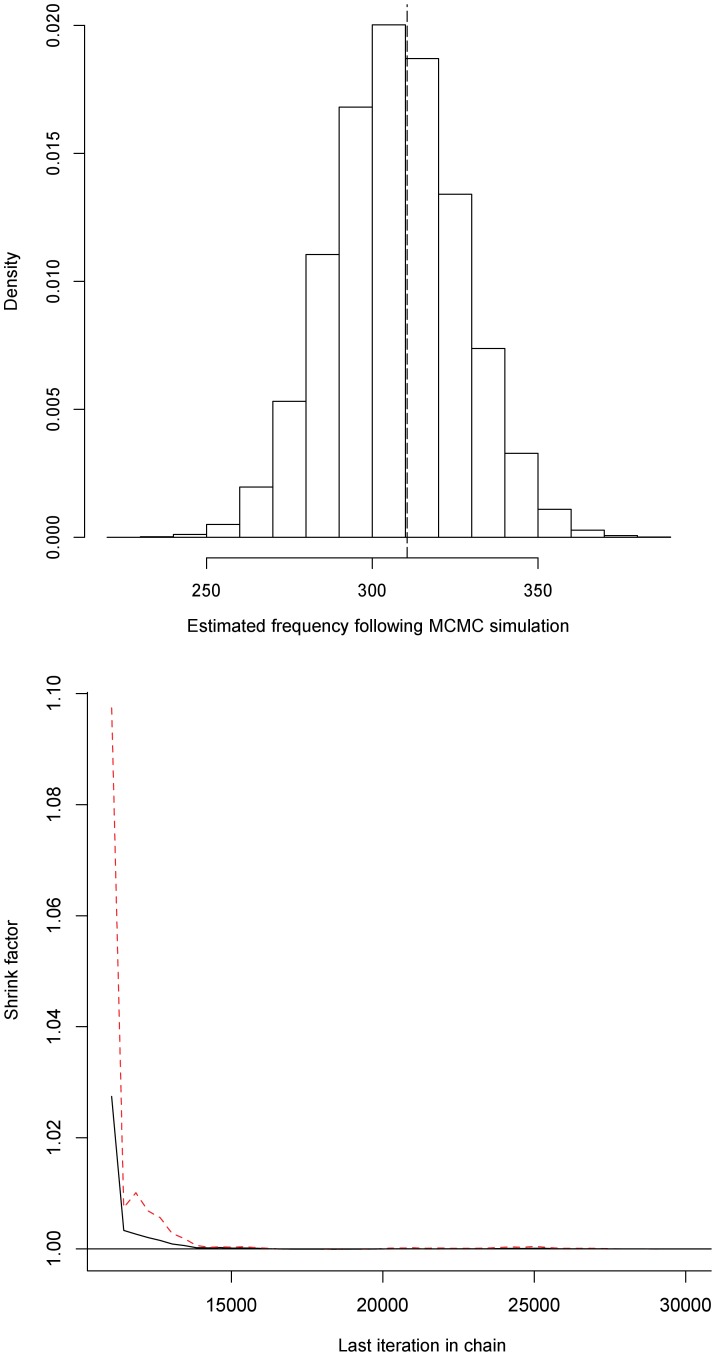
Results of Markov-Chain-Monte-Carlo (MCMC) simulations for the response profile ‘+++’. The estimated frequency is shown in the upper panel with the vertical dashed line representing the observed frequency. The associated Gelman-Rubin-Brooks plot demonstrating convergence during MCMC simulation is shown in the lower panel together with median (—) and 97.5% credible interval (----).

There have been few studies comparing diagnostic modalities in severe malaria and these have been conducted in sub-Saharan Africa where falciparum malaria is predominant [14], [15]. In Oceania, Asia and South America, *P. vivax* and mixed-species infections are increasingly recognized as important causes of severe disease [16]–[18]. A recent study of pediatric uncomplicated malaria in Papua New Guinea (PNG) showed that RDTs can guide treatment safely in an area of intense transmission of multiple *Plasmodium* species [19]. There are no equivalent data for severe malaria in this epidemiologic setting.

**Figure 2:**
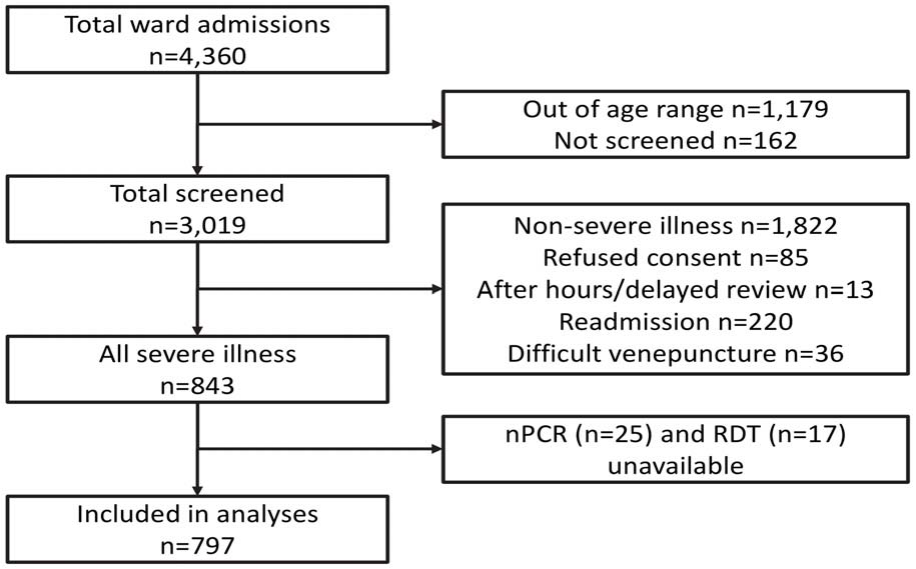
Consort diagram summarizing patient disposition after recruitment.

The application of Bayesian latent class models (LCM) is one approach to addressing the limitations of LM as a gold standard in studies of RDTs. LCM assume no gold standard and the true disease state (disease or no disease) for each individual is unknown. Bayesian approaches to LCM are increasingly used to validate diagnostic tests for infectious diseases without assuming a gold standard [20], [21], including as an epidemiological tool in the estimation of malaria prevalence [22] and in the evaluation of RDTs and other malaria diagnostic methods including LM for pooled data [5] and in children with and without fever [23]. In the latter study, the results of LCM analysis appeared robust, regardless of the choice of model and priors used [23].

**Table 1:** The observed and estimated frequencies with 95% credible intervals using MCMC for each response profile for all severe cases.

Response profiles of nPCR/LM/RDT results	Observed frequency	Median estimated frequency from MCMC	Credible Intervals (2.5^th^ –97.5^th^ centile)
– – –	279	276	240–314
– – +	35	35	21–53
– + –	2	3	0–9
– + +	7	7	2–17
+ – –	96	96	72–123
+ – +	54	54	36–75
+ + –	14	14	6–27
+ + +	310	308	270–346

**Table 2:** Diagnostic utility of nested PCR, light microscopy and malaria rapid diagnostic tests according to infecting *Plasmodium* species using a Bayesian latent class model that assumes no gold standard.

*Plasmodium* species	Model-derived malariaprevalence	Diagnostic modality	Sensitivity	Specificity	Negative predictive value	Positive predictive value
**Any** a	47.5 (43.7–51.3)	nPCR	97.6 (95.6–99.0)	74.8 (70.1–79.2)	97.2 (94.7–98.8)	77.8 (73.3–82.0)
		LM	87.4 (83.1–91.3)	99.1 (97.5–99.9)	89.7 (86.0–93.0)	98.9 (96.8–99.9)
		RDT	96.0 (93.4–97.9)	89.5 (85.6–92.7)	96.1 (93.5–98.0)	89.2 (85.1–92.6)
***P. falciparum*** ** only** b	42.9 (39.3–46.5)	nPCR	97.1 (94.8–98.8)	83.1 (79.1–86.6)	97.6 (95.4–98.9)	81.2 (76.7–85.2)
		LM	85.9 (81.4–89.8)	98.7 (97.0–99.6)	90.3 (87.0–93.2)	98.0 (95.6–99.4)
		RDT	98.0 (95.8–99.4)	89.2 (85.8–92.7)	98.4 (96.5–99.5)	87.8 (82.9–90.8)
**Mixed ** ***P. falciparum*** **/** ***P. vivax*** ** or ** ***P. vivax*** **infections** c	6.9 (4.5–11.2)	nPCR	91.8 (80.6–99.0)	89.7 (86.7–93.4)	99.3 (98.1–99.9)	39.4 (26.4–61.9)
		LM	77.5 (48.5–98.7)	99.8 (98.9–100)	98.4 (94.3–99.9)	96.2 (83.0–99.9)
		RDT	69.6 (55.8–81.8)	63.4 (60.0–67.0)	96.7 (93.1–98.3)	12.4 (7.8–19.3)
***P. vivax*** ** only** d	4.0 (2.6–6.2)	nPCR	89.8 (70.5–99.0)	95.4 (93.6–96.9)	99.6 (98.4–100)	44.4 (30.4–61.6)
		LM	80.9 (58.2–96.1)	99.0 (98.0–99.8)	99.2 (97.6–99.9)	77.4 (58.4–94.8)
		RDT	48.9 (30.9–67.8)	99.0 (98.1–99.6)	97.9 (96.1–98.9)	67.2 (45.7–85.8)

nPCR, nested polymerase chain reaction for *Plasmodium* species; LM, reference light microscopy of Giemsa-stained thick blood films; RDT, malaria rapid diagnostic test.

a Any *Plasmodium* species by nPCR, LM and either or both test lines positive by RDT.

b *P. falciparum* only by nPCR and LM; either PfHRP-2 line or both test lines positive by RDT.

c Mixed *P. falciparum/P. vivax* infection or single *P. vivax* infection by nPCR and LM; either aldolase line or both test lines positive by RDT.

d *P. vivax* only by PCR and LM; aldolase test line positive by RDT.

Data are shown as percentages and (95% credible intervals).

As part of a prospective study conducted in an area of PNG with hyperendemic transmission of both *P. falciparum* and *P. vivax*, we performed LM, RDTs and nested PCR (nPCR) to diagnose malarial illness in the setting of severely unwell, hospitalised children. Rather than assume that either LM or PCR was the gold standard, we developed a Bayesian LCM to determine the diagnostic performance of each test in the absence of a gold standard. The primary aim of the study was to determine whether the overall diagnostic performance of RDTs was equivalent to, or better than other diagnostic methods for severely ill children in an epidemiological setting where multiple Plasmodium species are transmitted. We also aimed to assess the diagnostic performance of RDTs according to infecting Plasmodium species and presenting clinical features.

**Table 3:** Diagnostic utility according to presenting clinical features for nested PCR, light microscopy and malaria rapid diagnostic tests using a Bayesian latent class model that assumes no gold standard.

Presenting clinicalfeatures	Model-derived malarial disease prevalence	Diagnostic modality	Sensitivity	Specificity	Negative predictive value	Positive predictive value
**Deep coma**	51.2 (38.7–63.0)	nPCR	89.6 (78.1–97.1)	66.8 (52.2–79.9)	86.7 (70.2–96.5)	73.0 (56.6–84.9)
		LM	77.4 (62.1–93.3)	96.4 (88.0–99.7)	81.0 (64.8–95.4)	95.6 (84.7–99.6)
		RDT	98.1 (90.9–99.9)	81.2 (64.5–95.2)	97.8 (88.8–99.9)	83.4 (65.3–96.4)
**Metabolic acidosis**	42.5 (30.8–54.6)	nPCR	95.2 (84.4–99.7)	82.4 (69.7–91.9)	95.9 (85.3–99.8)	79.9 (65.5–91.1)
		LM	94.2 (82.2–99.7)	88.4 (65.6–99.4)	95.4 (84.7–99.7)	86.0 (58.6–99.3)
		RDT	95.0 (84.1–99.7)	80.5 (67.6–90.5)	95.6 (84.9–99.8)	78.2 (63.6–89.6)
**Hyperlactatemia**	61.7 (50.6–71.5)	nPCR	97.4 (91.2–99.8)	60.5 (44.2–75.6)	93.6 (78.7–99.4)	80.0 (67.5–88.9)
		LM	84.8 (73.6–95.1)	97.2 (87.4–99.9)	79.6 (64.1–94.5)	98.0 (90.5–99.9)
		RDT	96.4 (89.3–99.7)	86.6 (69.5–97.1)	93.7 (81.2–99.4)	92.1 (79.3–98.4)
**Severe anemia**	73.4 (62.8–81.8)	nPCR	98.2 (93.6–99.9)	75.6 (54.4–92.5)	94.0 (78.4–99.6)	91.8 (80.8–97.9)
		LM	83.4 (73.9–92.9)	96.5 (84.5–99.9)	67.5 (49.7–87.1)	98.5 (92.8–99.9)
		RDT	97.0 (91.6–99.6)	63.4 (43.7–80.7)	88.4 (68.9–98.4)	88.1 (77.2–94.7)
**Respiratory distress**	22.8 (17.0–29.2)	nPCR	96.4 (88.4–99.6)	80.3 (73.4–86.2)	98.7 (95.6–99.9)	59.0 (46.6–70.5)
		LM	89.2 (75.9–98.3)	99.4 (96.9–100)	96.9 (92.5–99.6)	97.8 (88.6–99.9)
		RDT	88.1 (76.4–95.8)	94.2 (89.4–97.5)	96.4 (92.4–98.9)	81.7 (67.6–92.0)
**Mortality**	40.3 (26.7–54.5)	nPCR	90.2 (72.5–99.1)	95.5 (81.8–99.8)	93.6 (79.9–99.4)	93.2 (71.2–99.7)
		LM	47.3 (28.7–67.3)	97.4 (87.5–99.9)	73.3 (58.0–85.8)	92.6 (65.3–99.7)
		RDT	76.6 (57.3–91.5)	93.8 (80.5–99.6)	85.6 (71.0–95.3)	89.3 (66.4–99.3)

nPCR, nested polymerase chain reaction for *Plasmodium* species; LM, reference light microscopy of Giemsa-stained thick blood films; RDT, malaria rapid diagnostic test.

Data are shown as percentages and (95% credible intervals).

## Methods

### Study Site and Approvals

The present study was performed at Modilon Hospital in Madang Province on the north coast of mainland PNG, an area hyperendemic for both *P. falciparum* and *P. vivax* malaria [24]. Ethical approval for the study was obtained from the PNG Institute of Medical Research Institutional Review Board and the Medical Research Advisory Committee of the PNG Health Department. Written informed consent for participation was obtained from parent(s) or guardian(s) before recruitment.

### Patients

Between October 2006 and December 2009, all children aged 0.5–10 years admitted to Modilon Hospital, the provincial hospital to which the majority of children with severe illness are referred, were assessed for recruitment to an observational study of severe pediatric illness. Inclusion criteria included i) impaired consciousness (Blantyre Coma Score (BCS) ≤4, or ≤2 at 0.5, 1 or 6 hours after correction of hypoglycemia, a seizure or parenteral anticonvulsant therapy, respectively), ii) prostration (inability to sit/stand unaided), iii) multiple seizures, iv) hyperlactatemia (blood lactate >5.0 mmol/L), v) severe anemia (hemoglobin <50 g/L), vi) dark urine, vii) hypoglycemia (blood glucose ≤2.2 mmol/L), viii) jaundice, ix) respiratory distress (deep breathing, inter-costal in-drawing, sub-costal recession, persistent alar flaring, tracheal tug, and/or respiratory rate >60/minute), x) persistent vomiting, xi) abnormal bleeding, and/or xii) signs of shock. These criteria reflect the World Health Organization (WHO) definition of severe malarial illness [25]. Metabolic acidosis was defined as a plasma bicarbonate ≤12.2 mmol/L.

### Laboratory Procedures

A baseline venous blood sample was taken for LM and an ICT Malaria Combo Cassette Test MR2 (ICT Diagnostics, Brookvale, Australia). This RDT detects *P. falciparum* histidine-rich protein-2 (PfHRP-2) and aldolase from *P. falciparum* and non-falciparum malaria species, respectively. All RDTs were read by trained research nurses according to the manufacturer’s instructions. The presence of a single PfHRP-2 and aldolase line was considered diagnostic of mono-infection with *P. falciparum* and *P. vivax*, respectively. A positive test for both antigens indicated either *P. falciparum* alone or mixed infection. Giemsa-stained thick blood smears were examined independently by two skilled microscopists who were blinded to the RDT and nPCR results, with discrepancies adjudicated by a third microscopist. The peripheral blood parasitemia was quantified by counting the number of malaria parasites per 200 leucocytes assuming a peripheral blood leucocyte count of 8,000/µL. After parasite DNA extraction (QIAamp 96 DNA Blood Mini Kit, QIAGEN, Valencia, CA), we performed nPCR [26] to detect the presence of *Plasmodium* DNA and species.

Additional on-site tests comprised whole blood glucose (Hemocue, Ängelholm, Sweden) and lactate (Lactate Pro, Arkray, Japan), a full blood count (Coulter Ac·T diff, Beckman Coulter, Brea, USA) and blood culture. We also subsequently assayed plasma creatinine and bicarbonate (COBAS INTEGRA 800, Roche Diagnostics, Mannheim, Germany).

### Clinical Management

After recruitment, a standardized case report form that recorded demographic and medical data was completed. This included details of immunizations, past medical history and recent treatment with antimalarial drugs and antibiotics, as documented in each child’s hand-held medical record book. Clinical examination, inpatient management and follow-up were as described previously [18]. All severely ill children were treated with a course of intramuscular artemether, irrespective of parasitologic status [10], [18].

### Data Analysis

We generated LCMs using the statistical analysis package R [27] and the program JAGS [28]. In brief, all three diagnostic tests were incorporated into a multinomial model incorporating eight (2^3^) possible response profiles. These are denoted in the text and figures by a positive (+) or negative (–) result for each test. Outcomes of the diagnostic tests were assumed to be independent conditionally on the latent classes (disease or no disease). Formal model fitting was performed using Markov-Chain-Monte-Carlo (MCMC) simulations using a Gibb’s Sampler (R package rjags; code available on request) [28]. Non-informative priors were used and we used three chains. Each chain used a burn-in period of 10,000 iterations and was then run for another 20,000 iterations. Statistical inferences were based on the iterations after burn-in and, after 30,000 iterations, adequate convergence for the MCMC simulation was assessed visually using a Gelman-Rubin-Brooks plot (see Figure 1) and the Gelman diagnostic test. In addition, overall LCM fit was assessed using a Bayesian *P*-value comparing observed and calculated frequencies together with graphical representations for each response profile (see Figure 1).

## Results

### Patients

Of 4,360 children admitted to Modilon Hospital during the study period, 843 (19.3%) fulfilled the criteria for severe illness and were recruited. The median [IQR] age of this sub-group was 37 [18.3–45.2] months and 56.6% were males. All were of Melanesian racial background. There were 797 children (94.5%) with nPCR, LM and RDT results available and who were included in the present analyses (see Figure 2).

The clinical features of deep coma, metabolic acidosis, hyperlactatemia, severe anemia and respiratory distress were present in 104 (13.0%), 90 (11.3%), 109 (13.6%), 120 (15.1%) and 210 (26.3%) of children, respectively. The median [inter-quartile range] parasite density in the 299 children with *P. falciparum* by LM was 45,540 [4,266–125,215]/µL whole blood and that in the 44 children with *P. vivax* was 1,125 [171–5,451]/µL. Fifty-seven (7.2%) children died.

### Diagnostic Accuracy of RDTs, LM and nPCR

The observed and predicted frequencies for different response profiles along with MCMC estimations of 95% credible intervals (95% CI) for any malarial infection are shown in Table 1. The Bayesian *P*-value was 0.46, indicating good overall LCM fit and consistent with the assumption that the three diagnostic tests were independent conditional on the patient’s true disease status. The prevalence of malaria, and sensitivity, specificity, PPV and NPV’s for RDT (either test line positive), quality-assured LM (any species) and nPCR (any species positive) using a two-class LCM in which the true disease state for each individual was unknown are shown in Table 2.

Based on the LCM model, the estimated overall prevalence of any severe malaria was 47.5%. RDTs had a similar sensitivity (96.0%) and NPV (96.1%) to nPCR (97.6% and 97.2%, respectively). LM was the least sensitive test and had the lowest NPV (<90% in each case), but it had a higher specificity and PPV than the other two modalities (≥98.0% in each case). The diagnostic performances of all three tests for severe falciparum malaria were similar to those obtained for any severe malaria, consistent with the former group providing most of the cases (see Table 2). For non-falciparum severe malaria, RDTs were the least sensitive test (≤77.5%), especially for *P. vivax* mono-infections. RDTs also had the lowest NPVs in these two situations but the estimates were still >96%.

In some settings, both RDT and LM may be routinely and promptly available. To explore the diagnostic implications of this scenario, a further LCM was generated using RDT and LM results pooled as a single diagnostic modality with either test positive considered an overall positive result. The RDT/LM combination was compared to nPCR in a multinomial model with 2^2^ (four) response profiles for any severe malaria. The model-derived disease prevalence (56% [51–61%]) was higher than that in the LCM involving all three diagnostic modalities, while the sensitivity and NPV of nPCR increased to 99.8% [99.1–100] and 99.8 [98.8–100]), respectively. For the RDT/LM combination, sensitivity (94.4% [88.0–99.7]) and NPV (93.3% [85.0–99.7]) were between the values obtained for RDT and LM when they were considered as individual diagnostic modalities.

### Diagnostic Accuracy of RDTs, LM and nPCR by Presenting Clinical Features

The main presenting clinical features of the patients were deep coma (BCS≤2), metabolic acidosis (plasma bicarbonate <12.2 mmol/L), hyperlactatemia (blood lactate >5 mmol/L), severe anemia (hemoglobin <50 g/L) and respiratory distress (deep breathing, inter-costal in-drawing, sub-costal recession, persistent alar flaring, tracheal tug, and/or respiratory rate >60/minute). The sensitivity, specificity, NPV and PPV for each of these clinical features by diagnostic test using LCMs is shown in Table 3. LM had the lowest sensitivity and NPV for each clinical feature apart from respiratory distress. LM also had the lowest sensitivity for mortality.

## Discussion

The ideal diagnostic test is one that is easy to perform and interpret, can be widely deployed, and has high sensitivity and specificity. RDTs for malaria have the potential to provide valuable diagnostic information promptly and cost-effectively when a detailed initial clinical and laboratory assessment of a severely ill child is problematic, such as in a rural setting in a developing tropical country. There is increasing evidence that PfHRP-2-based RDTs are useful alternatives to LM for diagnosing severe falciparum malaria in African children, with sensitivities and NPVs relative to LM between 91–94% and 85–91%, respectively [14], [15]. The present data for a combined PfHRP-2-aldolase RDT extend this evaluation to a non-African area in which *P. falciparum* is the major cause of severe malarial illness but *P. vivax* is also a significant contributor.

The sensitivity and specificity of a malaria diagnostic test should be at least 95% and 90%, respectively, compared with expert LM [29]. The present LCM analyses show that the sensitivity of RDTs for any severe malaria and severe falciparum malaria in our PNG children was better than LM (≥96% compared with <90%) and that the relative specificities of RDTs were >90% those of LM. In the management of severely-ill children, a false negative result of a diagnostic test is the primary concern and an appropriate test thus requires high sensitivity and NPV. RDTs fulfilled these criteria for all severe malaria and severe falciparum malaria and were as good as nPCR in these two situations. Combining the RDT and LM test results attenuated the sensitivity and NPV of RDTs alone.

In the case of severe non-falciparum malaria, the type of RDT assessed in the present study did not perform as well as either nPCR or LM. The ICT MR2 test was selected prior to the publication of the first WHO testing rounds that are now in their fourth iteration and which demonstrated recently that the ICT MR2 had comparatively low sensitivity at *P. vivax* parasite densities <200/µL in uncomplicated cases [4]. Some newer RDTs have shown sensitivities of up to 100% for *P. vivax* under controlled conditions and would be better alternatives in future similar comparative diagnostic studies. However, it is important to note that, although the ICT MR2 RDT had the lowest sensitivity for non-falciparum malaria in our study, neither LM nor PCR had a sensitivity >95% in LCM models for mixed-species/*P. vivax* mono-infections or *P. vivax* mono-infections alone. Although these two groups contained a relatively small number of children, they have a mortality rate at least that of severe *P. falciparum* malaria [18]. Indeed, RDTs and especially LM had relatively low sensitivity for identifying children who were to die despite antimalarial therapy. RDTs had good sensitivity (≥95%) for the major presenting clinical features except respiratory distress, which is a more frequent feature of severe *P. vivax* and mixed-species infections than severe falciparum malaria in PNG children [18]. We did not find any differences in diagnostic performance by age (data not shown).

Extrapolating from the LCM model in the 797 children enrolled in which the malaria prevalence was 47.5% and the overall RDT sensitivity was 96%, there was a relatively small number of false negative RDT results (n = 16) that would include children infected with either or both *Plasmodium* species. In the case of *P. falciparum*, a false negative RDT could be due to either the prozone effect [30] or the deletion of PfHRP-2 and PfHRP-3 genes as found in South American isolates [31]. The prozone effect occurs when excess antigen blocks all sites for the colour change reaction and, in an African study, occurred in 1% of children with *P. falciparum* parasitemias >100,000/µL [14]. A false negative RDT in a child with *P. vivax* may reflect limited antigen availability and low test sensitivity at parasitemias <200/µL. The generally lower specificities of both RDTs and nPCR relative to LM are consistent with persistence of PfHRP-2 and nucleic acids in the absence of viable forms after successful prior treatment.

Although the present data show that none of the three diagnostic modalities assessed have optimal performance characteristics in the setting of hyperendemic mixed-species malaria transmission such as found in PNG and other parts of Oceania, as well as in Asia and South America, there appears a clear role for RDTs as a valuable point-of-care test that is at least equivalent to LM in diagnosing severe falciparum malaria. On-site field LM is often not as reliable as the expert LM used as a comparator in the present study, making a further argument for the use of RDTs as part of the initial evaluation of a severely ill child. However, diagnostic tests should only be part of this evaluation. Two important considerations are the prior probability of severe malaria and the presenting clinical features. In areas such as PNG and sub-Saharan Africa where falciparum malaria is the true diagnosis in up to 60% of severely ill children [14], [18], withholding empiric antimalarial therapy on the basis of a negative RDT would be inadvisable due to even a small risk of a false negative result. However, in a setting in which severe falciparum malaria accounted for <10% of hospitalizations, a negative RDT would give a <1% post-test probability that malaria was missed. If adequate clinical and laboratory monitoring were available, a decision not to give initial antimalarial therapy and pursue other diagnoses may be justifiable. A pertinent example of the value of adequate clinical assessment guiding treatment is respiratory distress which should signal the possibility of *P. vivax* malaria in an RDT-negative child.

With declining malaria mortality and hospitalizations in Africa [32] and beyond, and with the prospect of further improvements in the diagnostic sensitivity of RDTs, the utility and safety of RDT-based diagnostics algorithms as part of the management of severely ill children should be re-evaluated. In children with who have a negative RDT and a clear alternative diagnosis such as lobar pneumonia, measles or acute bacterial meningitis, withholding antimalarial therapy may be appropriate. Those with a positive RDT should be treated with antimalarial drugs but other diagnoses should still be considered. Until further evaluative studies are performed, severely ill children with a negative RDT and an indeterminate diagnosis should receive empiric antimalarial therapy as is usually recommended by local guidelines.
